# Prognostic Value of the Neo-Immunoscore in Renal Cell Carcinoma

**DOI:** 10.3389/fonc.2019.00439

**Published:** 2019-05-28

**Authors:** Congfang Guo, Hua Zhao, Yu Wang, Shuai Bai, Zizhong Yang, Feng Wei, Xiubao Ren

**Affiliations:** ^1^Department of Immunology, Tianjin Medical University Cancer Institute and Hospital, Tianjin, China; ^2^National Clinical Research Center for Cancer, Tianjin, China; ^3^Key Laboratory of Cancer Prevention and Therapy, Tianjin, China; ^4^Tianjin's Clinical Research Center for Cancer, Tianjin, China; ^5^Key Laboratory of Cancer Immunology and Biotherapy, Tianjin, China; ^6^Department of Emergency, Tianjin First Center Hospital, Tianjin, China; ^7^Department of Gastrointestinal Cancer Biology, Tianjin Medical University Cancer Institute and Hospital, Tianjin, China; ^8^Nankai University School of Medicine, Tianjin, China

**Keywords:** renal carcinoma, multiplex fluorescent immunohistochemistry, neo-immunoscore, risk stratification, prognosis

## Abstract

**Objective:** This study evaluated the prognostic value of the newly-built Immunoscore (neo-Immunoscore) in patients with renal cell carcinoma (RCC).

**Methods:** Eighty-two patients with RCC were enrolled in this study. Their 3- and 5-year survival rates and overall survival (OS) were evaluated. The clinicopathologic data of the 82 patients were collected and analyzed. CD3, CD4, CD8, CD45RO, Foxp3, tumor necrosis factor receptor type II (TNFR2), programmed death ligand-1 (PD-L1), CD68, programmed death-1 (PD-1), cytokeratin (CK), and indoleamine 2,3-dioxygenase (IDO) were separated into two panels and stained using multiplex fluorescent immunohistochemistry methods. An immunologic prediction model of RCC patients, the neo-Immunoscore (neo-IS), was constructed using a Cox regression model. For the prognostic prediction of RCC, the neo-IS with the immunoscore (IS) proposed by the Society for Immunotherapy of Cancer (SITC) were compared by receiver operator characteristic (ROC) curve analysis. Survivals between the neo-IS_low_ and neo-IS_high_ groups were analyzed using the Kaplan–Meier method. Multivariate Cox regression survival analysis was applied to analyze independent indicators.

**Results:** The Cox regression model allowed the establishment of a neo-IS based on three features: CD$3_{\text{CT}}^{+}$, CD4^+^Foxp3^+^CD45RO${}_{\text{CT}}^{+}$, and CD8^+^PD-$1_{\text{IM}}^{+}$. Compared to that of the IS proposed by the SITC, the neo-IS obtained a better prediction. The 3- and 5-year survival rates in neo-IS_high_ RCC patients were significantly higher than those in neo-IS_low_ RCC patients (94.7 vs. 77.4%, *P* = 0.035 and 94.7 vs. 64.5%, *P* = 0.002, respectively). The OS in the neo-IS_low_ group was significantly shorter than that in the neo-IS_high_ group (73 vs. 97 months, *P* = 0.000). In comparisons of the neo-IS with clinical pathological factors, we found that the risk stratification and neo-IS were independent factors for the prognosis of patients with RCC. Moreover, the OS rate of neo-IS_high_ RCC patients with low- and intermediate- risk was higher than that of neo-IS_low_ patients.

**Conclusion:** The newly-constructed IS model more precisely predicted the survival of patients with RCC and may supplement the prognostic value of risk stratification.

## Introduction

As one of the major urological cancers, renal cell carcinoma (RCC), which derives from renal tubular epithelial cells, constitutes ~3.8% of all cancers (1). RCC develops in about 295,000 people worldwide every year, with approximately 134,000 deaths due to RCC (2). The 5-year survival for kidney cancer is 74.5%; while 65.2% of kidney cancers are diagnosed at the local stage, the 5-year survival for localized kidney cancer is 92.6% (3). Even when curative surgery is performed for localized RCC patients, 20–30% experience recurrence or metastasis (1).

At present, the conventional prognostic prediction for RCC after radical nephrectomy is based on the American Joint Committee on Cancers (AJCC) pathological tumor-node-metastasis (TNM) classification system. Other pathological and clinical variables, including Fuhrman nuclear grade, necrosis, and Eastern Cooperative Oncology Group (ECOG) score have been implemented to improve the prognostication. Combining these tools together, the University of California Los Angeles Integrated Staging System (UISS) risk stratification provides a prognostic prediction for localized RCC (4). However, its predictive accuracy is still not comprehensive because it fails to incorporate the host immune response, which constitutes the major component of the defense system during tumor progression. In 2007, “immune contexture” including subtypes (CD3^+^CD8^+^, CD3^+^CD45RO^+^), functional orientation (Th1 cell-associated factors, cytotoxic factors, chemokines, cytokines, and adhesion molecules), density and location [tumor center [CT], invasive margin [IM] and the quality of tertiary lymphoid structures [TLS]] of immune infiltrating cells, were reviewed to show the important role of the immune system and a superior prognostic factor in cancers (5). Most studies have shown that high densities of CD3^+^ T cells, CD8^+^ cytotoxic T cells, CD45RO^+^ memory T cells, and granzyme B (GZMB) in both the CT and the IM are related to an improved overall survival (OS) (6). Because of the complexity of the immune contexture, the immunoscore (IS), which derived from the immune contexture and based on immune cell density, has been confirmed as a simple immune classification and a clinically useful prognostic marker in cancers. Due to the high background staining and loss of antigenicity of CD45RO and GZMB, Galon and other researchers proposed the use of two easy membrane stains, CD3 and CD8, both in CT and IM in the IS system proposed by the Society for Immunotherapy of Cancer (SITC) to initiate a task force to validate its use in standard clinical practice as a new approach for the classification of tumors (7). Several studies on the IS have focused on gastrointestinal tumors (8), as well as other cancers, such as lung cancer, liver cancer, and head and neck carcinoma (9, 10). However, few studies have assessed the prognostic factor of the IS in RCC; thus, our study explored the significance of the IS to predict the prognosis of patients with RCC.

The immunosuppressive microenvironment is well-known to play pivotal roles in tumor progression. CD4^+^Foxp3^+^ regulatory T cells (Tregs) are prototypical immunosuppressive cells that dampen excessive immune responses and maintain homeostasis of the immune system. Mechanisms of Treg-mediated suppression include secretion of immunosuppressive cytokines, cell-contact-dependent suppression and functional modification or killing of APC (11). RCC patients with increased Tregs had a poor clinical outcome (12). Subset of Treg expressing TNFR2 shows increased suppressive function relative to those that did not express tumor necrosis factor receptor type II (TNFR2) (13). CD45RO^+^ Treg (Memory Treg) cells have high immunosuppressive capacity and persist in the absence of antigen or low-level intermittent antigen exposure. Study of memory Treg cells in human has mainly focused on peripheral blood cells. It is important to study human memory Treg cells in tissues (14). On the other hand, macrophages are an important immune population. It can be subdivided into M1 and M2. M1 macrophages secrete pro-inflammatory cytokines and have a pro-inflammatory role. M2 macrophages have an anti-inflammatory role and favor tumor progression. Tumor associated macrophages (TAMs) are considered to be M2 macrophages according to widely accepted classification of macrophage. Increased infiltration of CD68^+^ TAMs in tumor tissues was correlated with recurrence in patients with RCC (15).

Tumor-induced immune suppression which is mediated by the programmed death-1 (PD-1) and its ligand, programmed death ligand 1 (PD-L1) make cancer cells evade the host immunity. PD-1, which is induced on effector T-cell, limits T-cell function in various peripheral tissues and conduce to tumor progression. Interaction of PD-1 with PD-L1 provides an immune escape mechanism for tumor cells by turning off cytotoxic T cells (16). In addition, indoleamine 2,3-dioxygenase (IDO), as a rate-limiting enzyme within a tryptophan-depleted microenvironment, represents some metabolism characteristics of RCC (17). However, few reports have included these markers in the IS system.

We incorporated these immunosuppressive factors into our newly-built IS model. In the present study, we incorporated TAM, Treg and its co-inhibitory molecules, CD8 or CD68 coexpressing PD-1 or IDO, CD3 coexpressing PD-L1 to build a novel immune feature-based score to predict the OS of RCC patients after nephrectomy and compared this new model to the IS proposed by the SITC.

Cytokeratin is a key tumor marker of epithelium origin; therefore, we analyzed its prognostic value for RCC and used cytokeratin to determine the expression of IDO in tumor or mesenchymal cells (18). As clear-cell renal cell carcinoma (ccRCC) is the most commonly encountered morphotype of RCC (19), we selected ccRCC patients to participate in our study.

## Methods

### Patients and Tissue Samples

Formalin-fixed paraffin-embedded specimens from tumor tissue of 82 RCC patients were included in this study and analyzed by multiplex fluorescent immunohistochemistry. The 82 patients were primary, biopsy-confirmed between January 2009 and January 2011 at Tianjin Medical University Cancer Institute and Hospital, Tianjin, China. These patients did not receive targeted therapy or immunotherapy before surgery. The study was approved by the Ethics Committee of Tianjin Cancer Institute and Hospital. Each patient gave written informed consent. According to the American Joint Committee on Cancer (AJCC)/Union for International Cancer Control (UICC) tumor–node–metastasis (TNM) staging system (4), the patients were classified as RCC subtypes I–II and III–IV. Based on the University of California Los Angeles Integrated Staging System (UISS) (4), the patients were categorized as low, intermediate, or high-risk. Neutrophil count, lymphocyte count, hemoglobin, platelet count, urea nitrogen, lactic dehydrogenase (LDH), beta-2 microglobulin (β2-MG) were obtained from peripheral blood samples before operation. NLR was calculated with neutrophil-to-lymphocyte ratio. The distributions of the patient demographic and clinical characteristics are listed in Table 1.

**Table 1:** Distributions of the estimated overall survival (OS) for every group of clinicopathological characteristics.

**Variables**	***N***	**Mean OS (months)**	***P***
Sex			0.176
Male	52	81	
Female	30	95	
Age (years)			0.120
< 60	59	90	
≥60	23	76	
Tumor size			0.281
≤ 7	71	89	
>7	11	72	
Tumor location			0.305
Left	40	82	
Right	42	89	
ECOG standard			0.013^*^
0	62	90	
≥1	20	69	
Fuhrman's grade			0.066
High	12	91	
Intermediate	54	90	
Low	16	71	
Stage			0.03^*^
I–II	62	91	
III–IV	20	73	
Risk			0.000^**^
Low	39	97	
Intermediate	31	87	
High	12	51	
Neutrophil			0.116
Normal	70	89	
< LLN or >ULN	12	77	
Lymphocyte			0.219
Normal	77	88	
< LLN or >ULN	5	72	
Hemoglobin			0.136
Normal	72	89	
< LLN or >ULN	10	72	
Platelets			0.001^**^
Normal	71	91	
< LLN or >ULN	11	59	
Urea nitrogen			0.332
Normal	68	88	
>ULN	14	78	
LDH			0.017^*^
Normal	77	89	
< LLN or >ULN	5	58	
β2-MG			0.524
Normal	67	89	
>ULN	15	85	

ECOG, Eastern Cooperative ONcology Group; LDH, lactic dehydrogenase; LLN, lower limit of normal; ULN, upper limit of normal; β2-MG, beta-2 microglobulin; OS, overall survival.

* P < 0.05;

** *P < 0.01*.

### Multiplex Fluorescent Immunohistochemistry and Multispectral Imaging

We selected 11 markers for multiplex fluorescent immunohistochemistry (IHC) staining to detect pan T cells (CD3), helper T cells (CD4), memory T cells (CD45RO), forkhead box P3 (Foxp3), TNFR2, and programmed death ligand-1 (PD-L1) by seven-color IHC and cytotoxic T cells (CD8), macrophages (CD68), programmed death-1 (PD-1), cytokeratin (CK), and indoleamine 2,3-dioxygenase (IDO) for six-color IHC. We analyzed the expression levels of single-stained cells, double-stained cells (CD3^+^PD-L1^+^, CD8^+^PD-1^+^, CD68^+^PD-1^+^, CD8^+^IDO^+^, CD68^+^IDO^+^, and CD4^+^FoxP3^+^), and triple-stained cells (CD4^+^Foxp3^+^CD45RO^+^ and CD4^+^Foxp3^+^TNFR2^+^) in the CT and IM of RCC specimen slides.

Multiplex fluorescent staining was obtained using Opal-7-Color Manual IHC Kit (NEL81001KT, PerkinElmer). The slides were deparaffinized in xylene and rehydrated in ethanol. Antigen retrieval was performed in AR 9.0 Buffer with microwave treatment (MWT). The tissue sections were covered with PerkinElmer Antibody Diluent blocking buffer and incubated for 10 min at room temperature. Primary antibodies for PD-L1 (66248-1-Ig Proteintech, 1:2000) were incubated in a refrigerator at 4°C overnight. The following day, the slides were incubated in Polymer HRP Ms+Rb for 10 min at room temperature. Visualization of PD-L1 was amplificated using Opal 520 TSA Plus (1:100). Then, the slides were antigen retrieved in AR 6.0 buffer with heated MWT. The preceding steps, including blocking, primary antibody incubation, introduction of HRP, signal amplification, and antibody stripping via microwave treatment were repeated until all targets of interest, including CD45RO (55618S CST, 1:2000), Foxp3 (MAB8214 R&D, 1:500), CD4 (ab133616, 1:500), TNFR2 (ab109322, 1:400), and CD3 (MA5-14524 Invitrogen, 1:300) were detected using a corresponding Opal fluorophore (Opal 540, Opal 570, Opal620, Opal650, or Opal690). The cell nuclei were finally stained with 4′,6-diamidino-2-phenylindole solution and the slides were cover-slipped with mounting medium. PD-1 (ab137132, 1:800), CD68 (ZM-0464, ZSGB-BIO, 1:200), CD8 (ab4055, 1:400), IDO (ab55305, 1:800), and CK (ZM-0069, ZSGB-BIO, 1:500) were detected using a series of Opal fluorophores (Opal520, Opal 540, Opal 570, Opal620, or Opal690) in another six-color IHC.

Visualization of the seven-color Opal slides was performed using a Mantra Quantitative Pathology Imaging System (PerkinElmer), which captured the fluorescent spectra at 20-nm wavelength intervals from 420 to 720 nm with the same exposure times to produce combined single-stack images. Images of single-stained and unstained tissue were used to extract the spectrum of each fluorophore and of tissue autofluorescence, separately, and to create the spectrum required for multispectral unmixing, which was performed using InForm image analysis software (PerkinElmer).

Five representative fields were selected to scan the CT and IM, respectively. The density was recorded as the number of positive cells per unit tissue surface area at × 200 magnification (1 mm). The nucleated stained cells in each area were quantified and expressed as the number of cells per area.

### Statistical Analysis

The OS was calculated from the time of surgery until death with patients still alive censored at the time of their last contact in January 2018. The binary variables were analyzed by the Kaplan-Meier method, while triad or continuous variables were assessed by the Cox approach. A Cox proportional hazards model was used in multivariable analyses using the Forward-LR method with a significance level of 0.20 for entering and removing variables, resulting in a three-feature-based model to assess the prognostic value. The correlations of cell densities with different biomarkers were evaluated by Spearman's correlation analysis to determine why some variables were included or removed from the model. Receiver operator characteristic (ROC) curve analysis was used to compare the neo-IS to the IS proposed by the SITC. *P* < 0.05 in two-sided tests indicated statistical significance. All calculations were performed using IBM SPSS Statistics for Windows, version 22.0.

## Results

### Patient Characteristics and Neo-IS_RCC_ Construction

**Figure 1:**
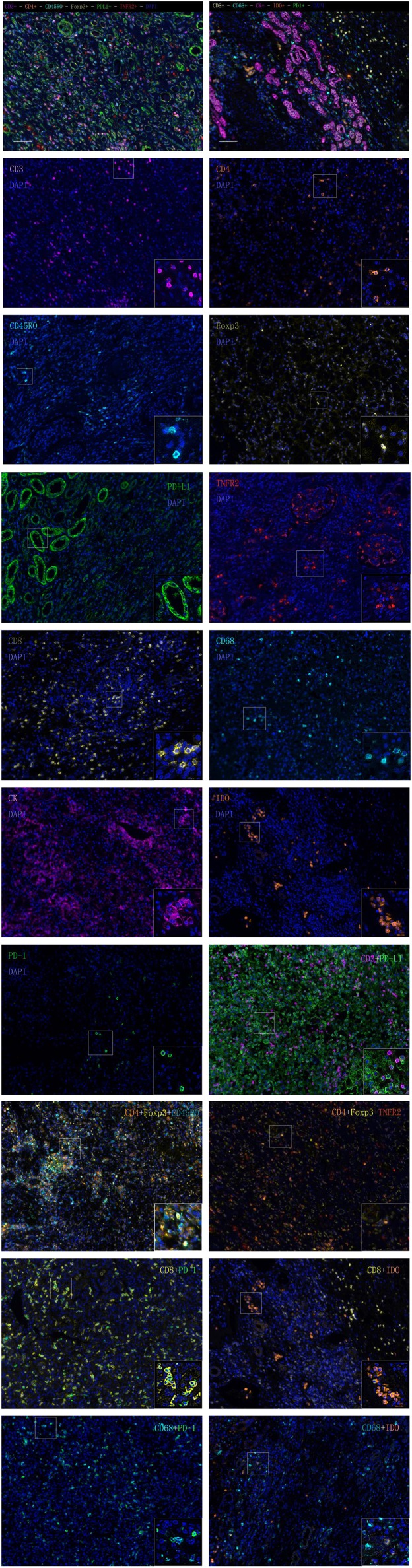
Multiplex fluorescent immunohistochemistry staining.

**Figure 2:**
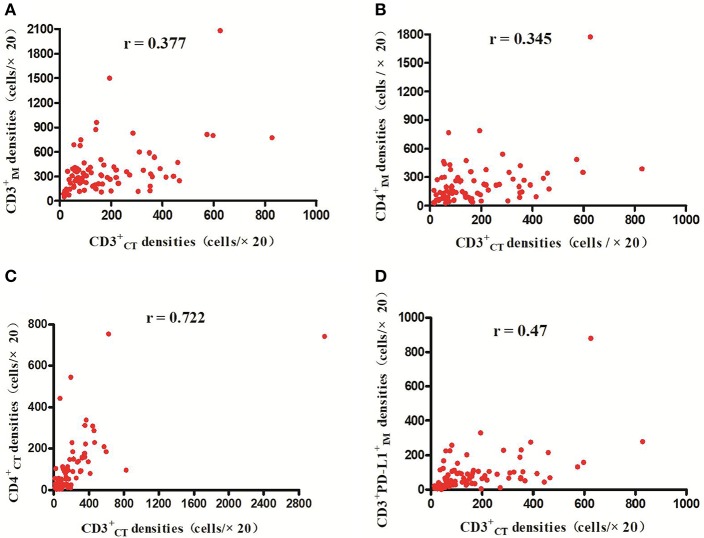
Positive correlations between CD$3_{\text{CT}}^{+}$ and CD$3_{\text{IM},}^{+}$ CD$4_{\text{IM},}^{+}$ CD$4_{\text{CT},}^{+}$ and CD3^+^PD-L$1_{\text{IM}}^{+}$ cell densities, respectively (*p* < 0.01) **(A–D)**.

**Figure 3:**
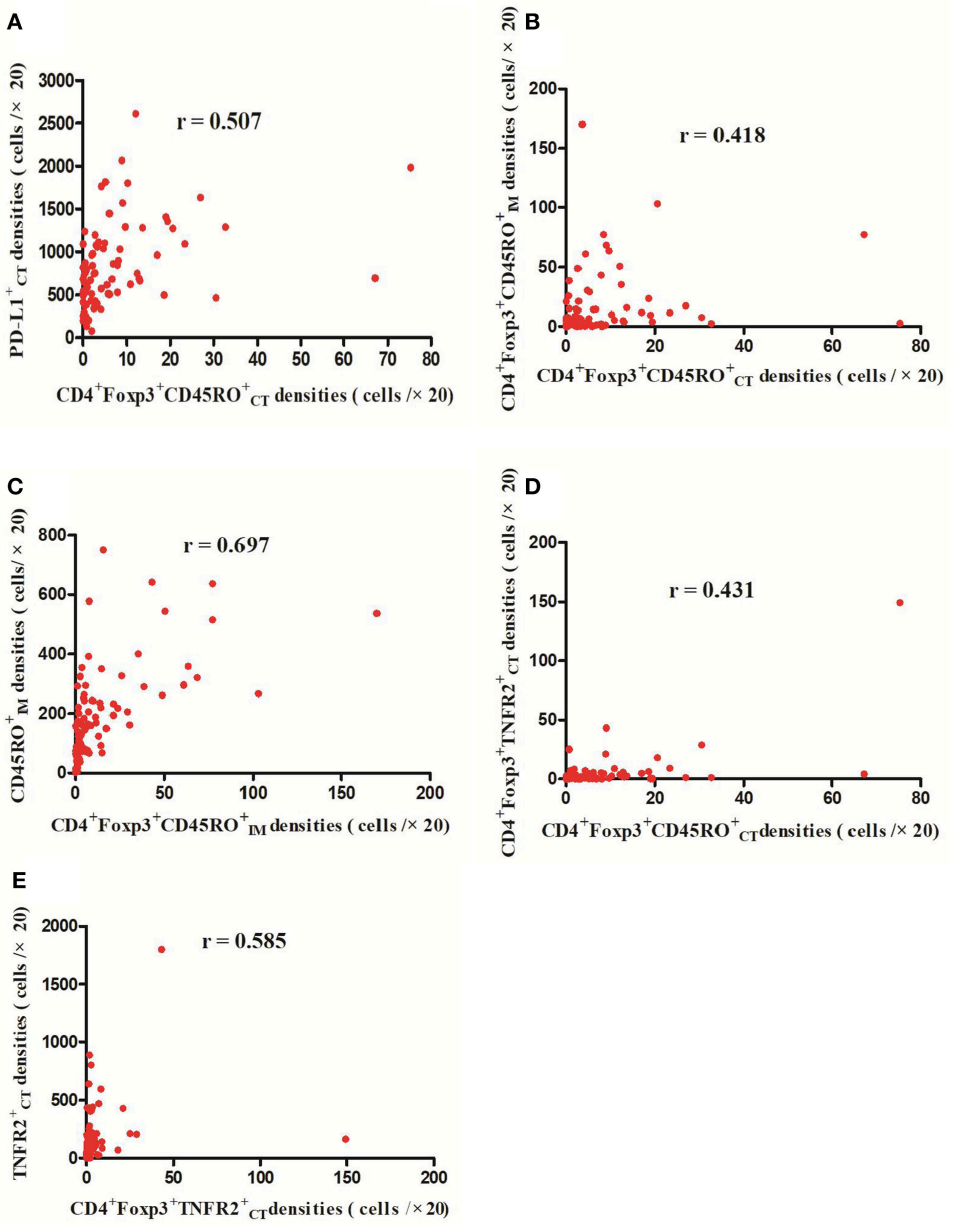
Positive correlations between CD4^+^Foxp3^+^CD45RO${}_{\text{CT}}^{+}$ and PD-L$1_{\text{CT},}^{+}$ CD4^+^Foxp3^+^CD45RO${}_{\text{IM},}^{+}$ and CD4^+^Foxp3^+^TNFR$2_{\text{CT}}^{+}$ cell densities, respectively (*p* < 0.01) **(A,B,D)**; Meanwhile, CD4^+^Foxp3^+^CD45RO${}_{\text{IM}}^{+}$ cell densities were positively related with CD45RO${}_{\text{IM}}^{+}$ cell densities **(C)** (*p* < 0.01) and CD4^+^Foxp3^+^TNFR$2_{\text{CT}}^{+}$ cell densities were positively correlated with TNFR$2_{\text{CT}}^{+}$ cell densities (*p* < 0.01) **(E)**.

**Figure 4:**
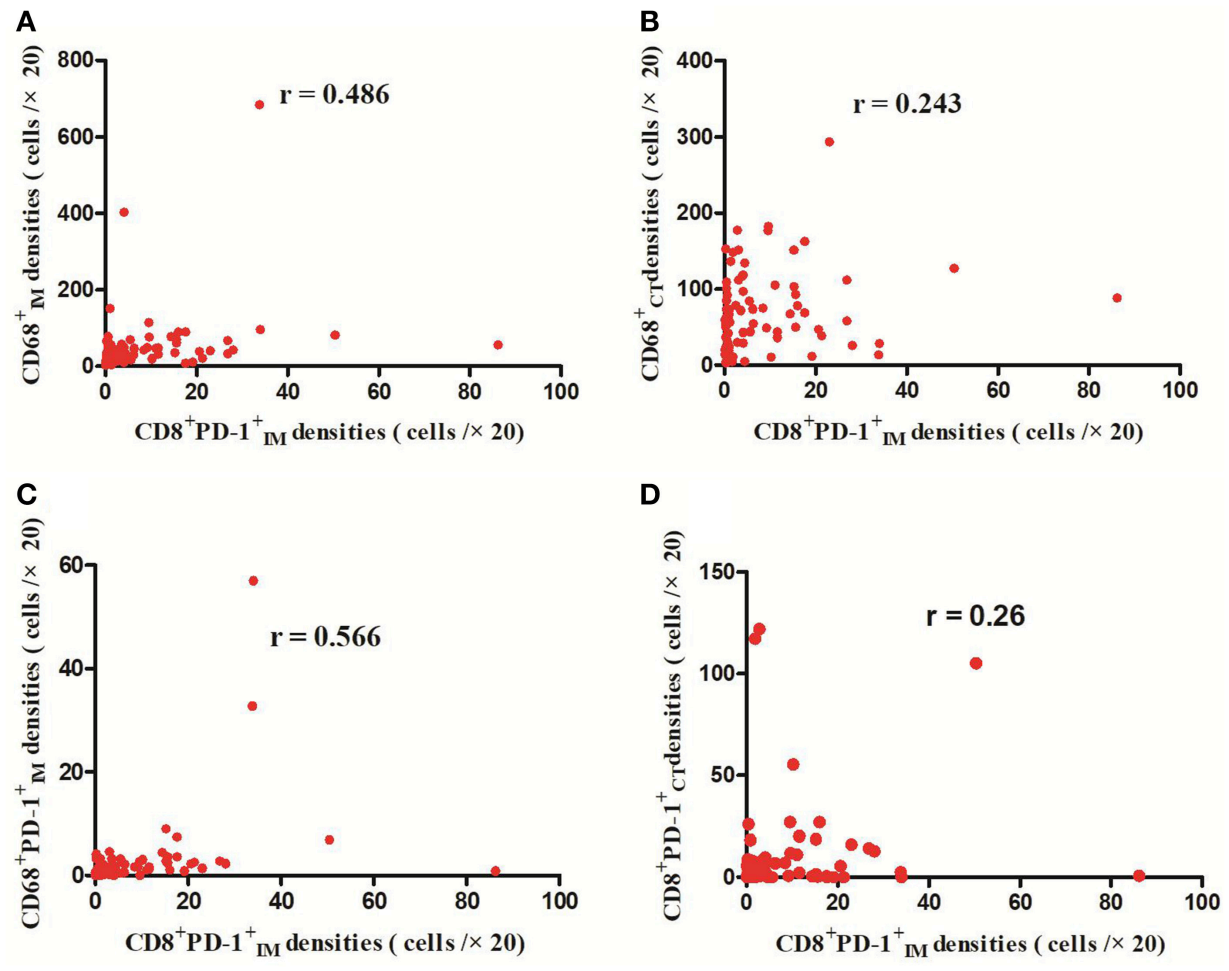
Positive correlations between CD8^+^PD-$1_{\text{IM}}^{+}$ and CD$68_{\text{IM}}^{+}$, CD$68_{\text{CT}}^{+}$, CD68^+^PD-$1_{\text{IM}}^{+}$, and CD8^+^PD-$1_{\text{CT}}^{+}$ cell densities (*p* < 0.01) **(A–D)**.

The detailed clinicopathological characteristics of the 82 RCC patients are shown in Table 1. We identified a dominant cluster in multiplex fluorescent IHC analysis including 15 features out of a total of 19 biomarkers in the CT and IM (CD$3_{\text{CT}}^{+}$, CD3^+^PD-L$1_{\text{IM}}^{+}$, CD68_IM_, *P* < 0.05) (PD-L1_CT_, CD3_IM_, CD4_IM_, CD4_CT_, CD45RO_IM_, CD4^+^Foxp3^+^CD45RO${}_{\text{CT}}^{+}$, TNFR2_CT_, CD4^+^Foxp3^+^TNFR$2_{\text{CT}}^{+}$, CD68^+^PD-$1_{\text{IM}}^{+}$, CD8^+^PD-$1_{\text{IM}}^{+}$, CD8^+^PD-$1_{\text{CT}}^{+}$, CD68_CT_, *P* < 0.2) (Table 2). The multispectral images of each biomarker were recorded (Figure 1). The correlations with 15 features were analyzed by Spearman analysis, which resulted in some features being included and removed from the multivariable Cox regression. The CD$3_{\text{CT}}^{+}$ cell density was positively correlated to those of CD$3_{\text{IM}}^{+}$, CD$4_{\text{IM}}^{+}$, CD$4_{\text{CT}}^{+}$, and CD3^+^PD-L$1_{\text{IM}}^{+}$ cells (*P* < 0.01) (Figures 2A–D). The CD4^+^Foxp3^+^CD45RO${}_{\text{CT}}^{+}$ cell density was positively correlated to those of PD-L$1_{\text{CT}}^{+}$, CD4^+^Foxp3^+^CD45RO${}_{\text{IM}}^{+}$, and CD4^+^Foxp3^+^TNFR$2_{\text{CT}}^{+}$ cells (*P* < 0.01) (Figures 3A,B,D). In addition, the CD4^+^Foxp3^+^CD45RO${}_{\text{IM}}^{+}$ cell density was positively correlated to that of CD45RO${}_{\text{IM}}^{+}$ cells (*P* < 0.01) (Figure 3C), while CD4^+^Foxp3^+^TNFR$2_{\text{CT}}^{+}$ cell density was positively correlated with that of TNFR$2_{\text{CT}}^{+}$ cells (*P* < 0.01) (Figure 3E). The CD8^+^PD-$1_{\text{IM}}^{+}$ cell density was positively correlated to those of CD$68_{\text{IM}}^{+}$, CD$68_{\text{CT}}^{+}$, CD68^+^PD-$1_{\text{IM}}^{+}$, and CD8^+^PD-$1_{\text{CT}}^{+}$ cells (*P* < 0.01) (Figures 4A–D).

**Table 2:** Univariate analysis of 82 RCC patients on all biomarkers and overall survival.

**Parameters**	**OS**		
	**Hazard ratio**	**95%CI**	***P***
PD-L$1_{\text{IM}}^{+}$	1.001	0.999–1.002	0.454
PD-L$1_{\text{CT}}^{+}$	1.001	1.000–1.001	0.115^*^
CD$3_{\text{IM}}^{+}$	0.998	0.995–1.001	0.193^*^
CD$3_{\text{CT}}^{+}$	0.992	0.986–0.998	0.013^**^
CD$4_{\text{IM}}^{+}$	0.996	0.992–1.001	0.127^*^
CD$4_{\text{CT}}^{+}$	0.995	0.988–1.002	0.149^*^
CD45RO${}_{\text{IM}}^{+}$	0.997	0.992–1.001	0.134^*^
CD45RO${}_{\text{CT}}^{+}$	0.999	0.993–1.004	0.677
Foxp$3_{\text{IM}}^{+}$	1.000	0.999–1.002	0.684
Foxp$3_{\text{CT}}^{+}$	1.000	0.998–1.002	0.776
CD4^+^Foxp$3_{\text{IM}}^{+}$	1.002	0.994–1.009	0.648
CD4^+^Foxp$3_{\text{CT}}^{+}$	1.004	0.991–1.018	0.528
CD4^+^Foxp3^+^CD45RO${}_{\text{IM}}^{+}$	0.993	0.970–1.017	0.591
CD4^+^Foxp3^+^CD45RO${}_{\text{CT}}^{+}$	1.018	0.992–1.044	0.182^*^
TNFR$2_{\text{IM}}^{+}$	1.000	0.998–1.003	0.679
TNFR$2_{\text{CT}}^{+}$	1.001	1.000–1.002	0.104^*^
CD4^+^Foxp3^+^TNFR$2_{\text{IM}}^{+}$	1.004	0.995–1.013	0.365
CD4^+^Foxp3^+^TNFR$2_{\text{CT}}^{+}$	1.012	1.000–1.025	0.059^*^
CD3^+^PD-L$1_{\text{IM}}^{+}$	0.988	0.977–0.999	0.039^**^
CD3^+^PD-L$1_{\text{CT}}^{+}$	0.996	0.989–1.003	0.223
IDO${}_{\text{IM}}^{+}$	1.001	0.999–1.004	0.331
IDO${}_{\text{CT}}^{+}$	1.000	0.999–1.001	0.964
PD-$1_{\text{IM}}^{+}$	1.001	0.987–1.014	0.918
PD-$1_{\text{CT}}^{+}$	0.996	0.980–1.012	0.616
CD68^+^PD-$1_{\text{IM}}^{+}$	1.026	0.992–1.061	0.140^*^
CD68^+^PD-$1_{\text{CT}}^{+}$	0.852	0.623–1.166	0.318
CD8^+^PD-$1_{\text{IM}}^{+}$	1.022	0.998–1.047	0.071^*^
CD8^+^PD-$1_{\text{CT}}^{+}$	0.925	0.831–1.030	0.157^*^
CK${}_{\text{IM}}^{+}$	1.000	0.998–1.001	0.624
CK${}_{\text{CT}}^{+}$	0.930	0.998–1.002	0.930
CD$8_{\text{IM}}^{+}$	1.002	0.999–1.004	0.267
CD$8_{\text{CT}}^{+}$	0.998	0.994–1.002	0.316
CD$68_{\text{IM}}^{+}$	1.003	1.000–1.006	0.033^**^
CD$68_{\text{CT}}^{+}$	0.992	0.980–1.003	0.162^*^
CD8^+^IDO${}_{\text{IM}}^{+}$	1.015	0.988–1.042	0.288
CD8^+^IDO${}_{\text{CT}}^{+}$	0.995	0.976–1.014	0.604
CD68^+^IDO${}_{\text{IM}}^{+}$	1.002	0.951–1.055	0.947
CD68^+^IDO${}_{\text{CT}}^{+}$	0.989	0.944–1.036	0.989

* P < 0.2;

** *P < 0.05*.

**Figure 5:**
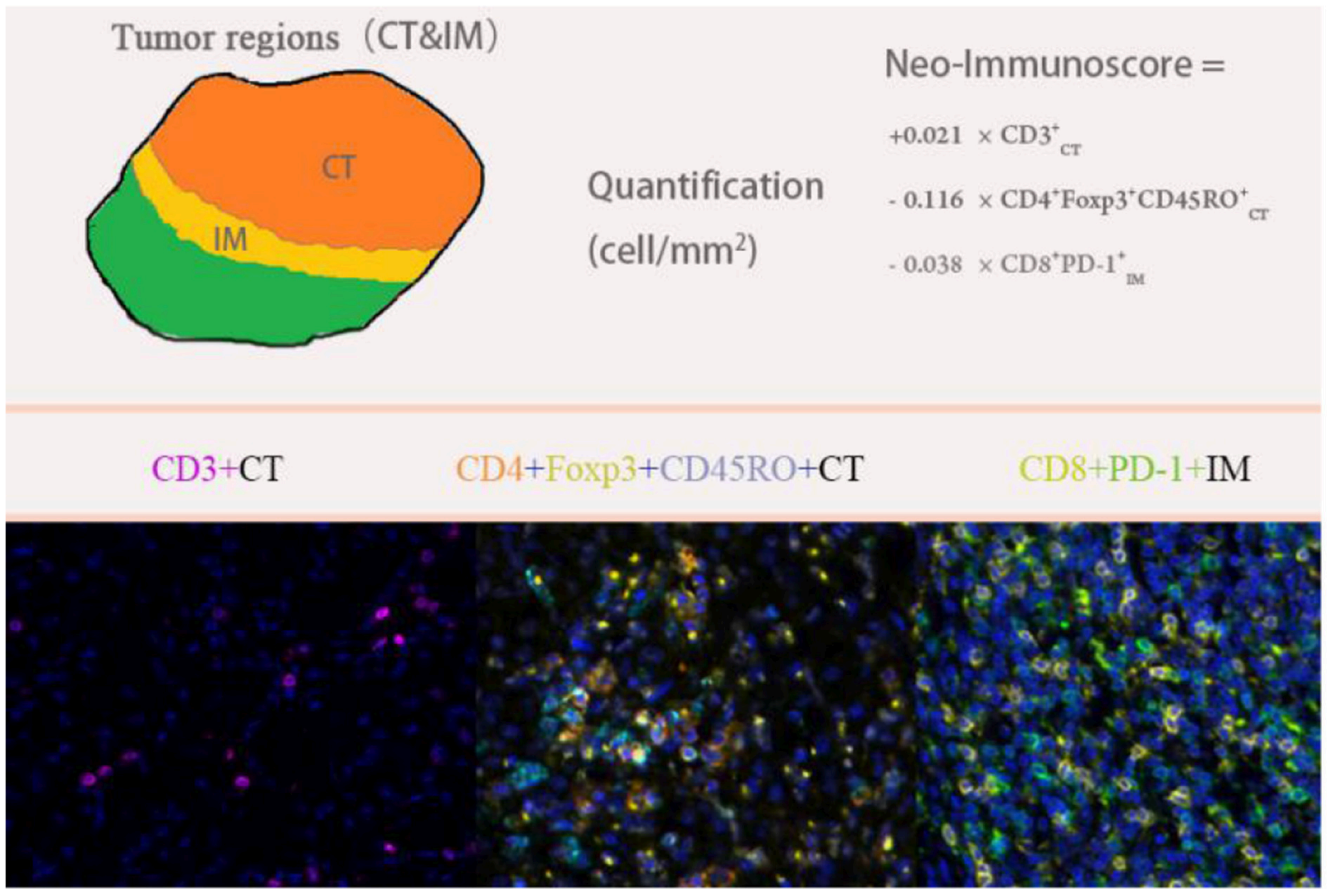
Neo-Immunoscore definition and methodology.

We used multivariable Cox regression analysis to construct the neo-IS based on the levels of three features, where neo-IS = -PI (Prognostic Index) = 0.021 × CD$3_{\text{CT}}^{+}$ density−0.116 × CD4^+^Foxp3^+^CD45RO${}_{\text{CT}}^{+}$ density−0.038 × CD8^+^PD-$1_{\text{IM}}^{+}$ density (Figure 5). The multivariable Cox model suggested that CD$3_{\text{CT}}^{+}$ cells played a protective role in the prognosis of RCC patients and that CD4^+^Foxp3^+^CD45RO${}_{\text{CT}}^{+}$ T and CD8^+^PD-$1_{\text{IM}}^{+}$ T cells were negative factors for the prognosis of RCC patients (Table 3).

**Table 3:** Multivariable analysis of 82 RCC patients on the selected biomarkers and overall survival.

**Parameters**	**OS**		
	**Hazard ratio**	**95%CI**	***P***
CD3_CT_	0.979	0.969–0.990	0.000
CD4^+^Foxp3^+^CD45RO${}_{\text{CT}}^{+}$	1.123	1.061–1.188	0.000
CD8^+^PD-$1_{\text{IM}}^{+}$	1.039	1.004–1.074	0.027

### Comparison of the IS Proposed by the SITC and the Neo-IS

**Figure 6:**
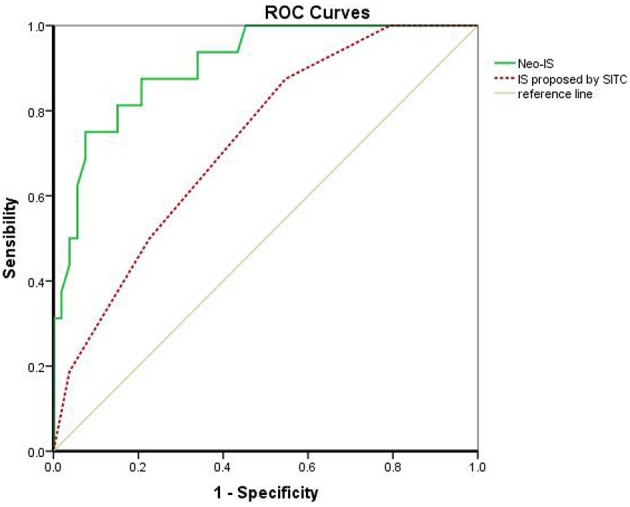
Comparison of the IS proposed by the SITC and the neo-IS. IS proposed by SITC calculated with CD3 and CD8 by CT/ IM. Neo-IS = 0.021 × CD$3_{\text{CT}}^{+}$ density−0.116 × CD4^+^Foxp3^+^CD45RO${}_{\text{CT}}^{+}$ density−0.038 × CD8^+^PD-$1_{\text{IM}.}^{+}$ Density.

The IS proposed by the SITC is based on the enumeration of two lymphocyte populations (CD3 or CD8) quantified within the CT and IM. It provides a scoring system ranging from IS 0 (I0), with low densities of both cell populations in both regions, to IS 4 (I4), with high densities of both cell types in both regions. Multivariable Cox regression survival analysis in the present study revealed that RCC patients with a higher IS proposed by the SITC had a better survival than that in those with lower IS (β −0.610, hazard ratio 0.543, 95%CI 0.343–0.860, *P* < 0.01). However, the AUC of the neo-IS was higher than that of the IS proposed by the SITC by ROC curve analysis (AUC 0.906 vs. 0.725, *P* < 0.01) (Figure 6).

### Prognoses of RCC Patients With High and Low Neo-IS

The 3- and 5-year survival rates of the 82 RCC patients were 87.1 and 81.2%, respectively. The OS of these patients was 87 months (95% CI 80–94 months).

The 3- and 5-year survival rates in the neo-IS_high_ RCC patients were significantly higher than those in the neo-IS_low_ patients (94.7 vs. 77.4%, *P* = 0.035 and 94.7 vs. 64.5%, *P* = 0.002, respectively).

**Figure 7:**
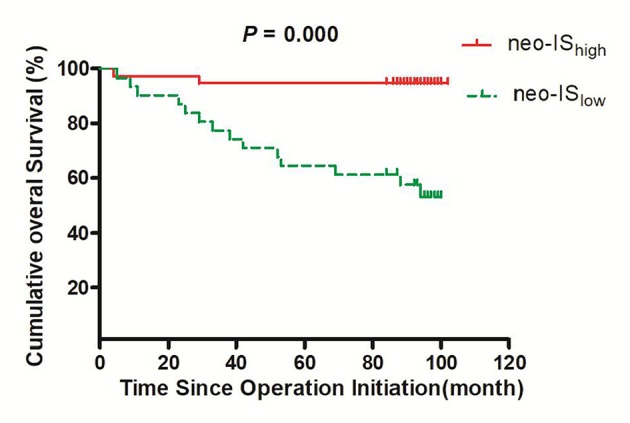
Survival curves for overall survival (OS) in the neo-IS_low_ and neo-IS_high_ groups.

The OS in neo-IS_low_ patients was significantly shorter than that in neo-IS_high_ patients (73 vs. 97 months, *P* = 0.000) (Figure 7).

### Prognoses of RCC Patients With Different Clinicopathological Characteristics

**Figure 8:**
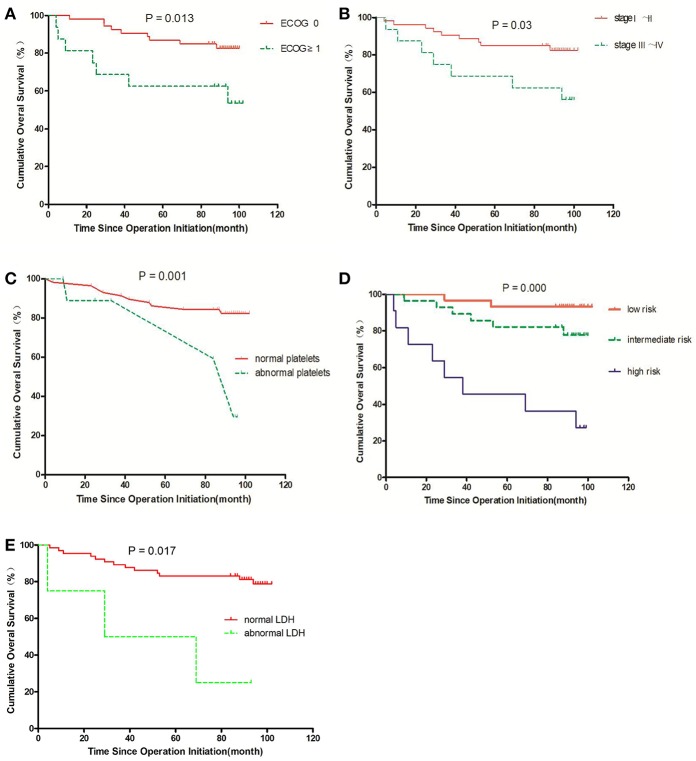
Survival curves for overall survival (OS) in RCC patients; an event is defined as death from any cause in OS. The comparisons are as follows: **(A)** ECOG status; **(B)** stage; **(C)** normal or abnormal platelets; **(D)** risk group; **(E)** normal or abnormal LDH.

We compared the prognoses of 82 RCC patients according to sex, age, tumor size, tumor location, ECOG, histology, stage, risk stratification, *n*eutrophil, lymphocyte, LDH, hemoglobin, platelets, urea nitrogen, and β2-MG by univariate analysis (Table 1). The OS of patients with ECOG 0 was longer than that in those with ECOG ≥ 1 (*P* = 0.013) (Figure 8A). The OS of patients with stage I–II disease was longer than that in those with stage III–IV disease (*P* = 0.030) (Figure 8B). Patients with abnormal platelets had a significantly shorter OS than that in those with normal platelets (*P* = 0.001, Figure 8C). The diverse risk groups had significantly different OS (*P* = 0.000, Figure 8D). Patients with abnormal LDH had a significantly shorter OS than that in those with normal LDH (*P* = 0.017, Figure 8E). NLR had no prognostic value in patients with different NLR (*P* = 0.324 > 0.05).

### Correlations Between Neo-IS and Clinicopathological Characteristics

There were no correlations between neo-IS and neutrophil (*P* = 0.285 > 0.05), lymphocyte (*P* = 0. 721 > 0.05), platelets (*P* = 0.132 > 0.05), hemoglobin (*P* = 0.054 > 0.05), urea nitrogen (*P* = 0.306 > 0.05), β2-MG (*P* = 0.150 > 0.05), LDH (*P* = 0.116 > 0.05), or NLR (*P* = 0.245 > 0.05).

The neo-IS was also not related with Fuhrman's grade (*P* = 0.111 > 0.05), tumor size (*P* = 0.626 > 0.05), tumor location (*P* = 0.716 > 0.05) *or* risk stratification (*P* = 0.087 > 0.05). However, neo-IS was negatively correlated to staging (r = −0.226, *P* = 0.041 < 0.05) and ECOG (r = −0.223, *P* = 0.044 < 0.05).

### Multiple-Factor Analysis of the Prognostic Factors in Patients With RCC

Multivariable Cox regression analysis revealed that risk stratification was a negative prognostic factor in RCC patients (β = 2.422, hazard ratio = 11.263, 95%CI: 1.610–78.82, *P* = 0.015 < 0.05) and that the neo-IS (β = −0.810, hazard ratio = 0.445, 95%CI: 0.284–0.696, *P* = 0.000 < 0.01) was a predictive factor in RCC patients.

There was no correlation between the neo-IS and risk stratification (*P* = 0.087 > 0.05). However, risk stratification was positively correlated to platelets (r = 0.237, *P* < 0.05), ECOG (r = 0.281, *P* < 0.05), and staging (r = 0.573, *P* < 0.01); thus, staging, ECOG, and platelets removed from the multivariable Cox regression.

**Figure 9:**
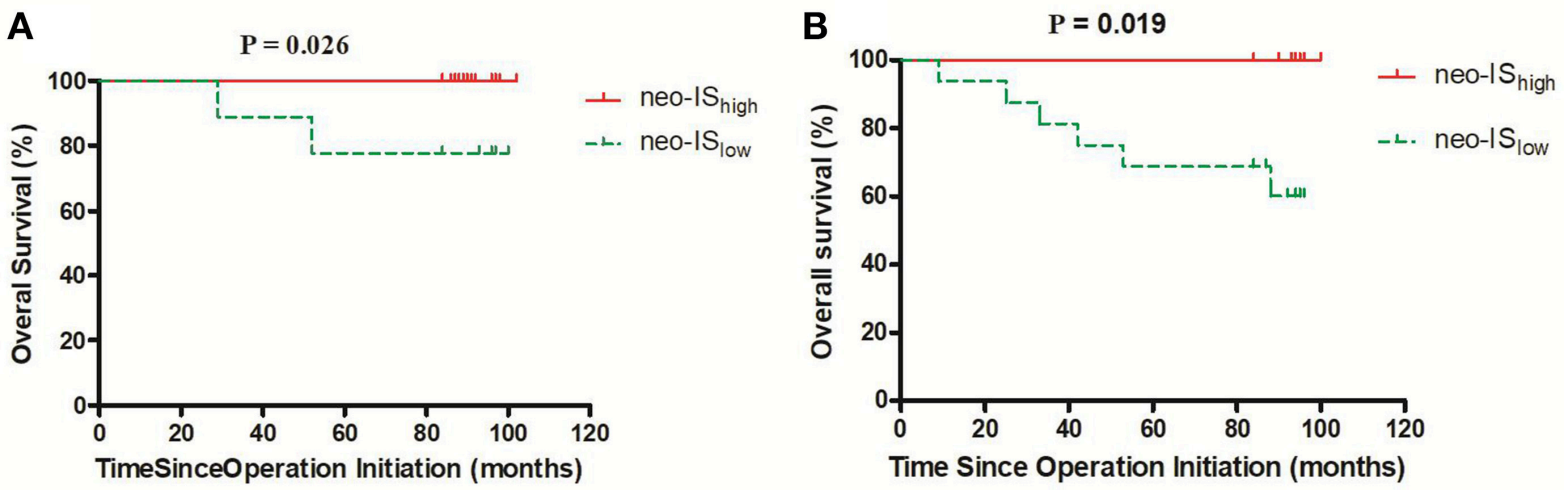
Survival curves for overall survival (OS) in RCC patients with low- and intermediate-risk stratification. **(A)** Low-risk stratification. **(B)** Intermediate-risk stratification.

**Figure 10:**
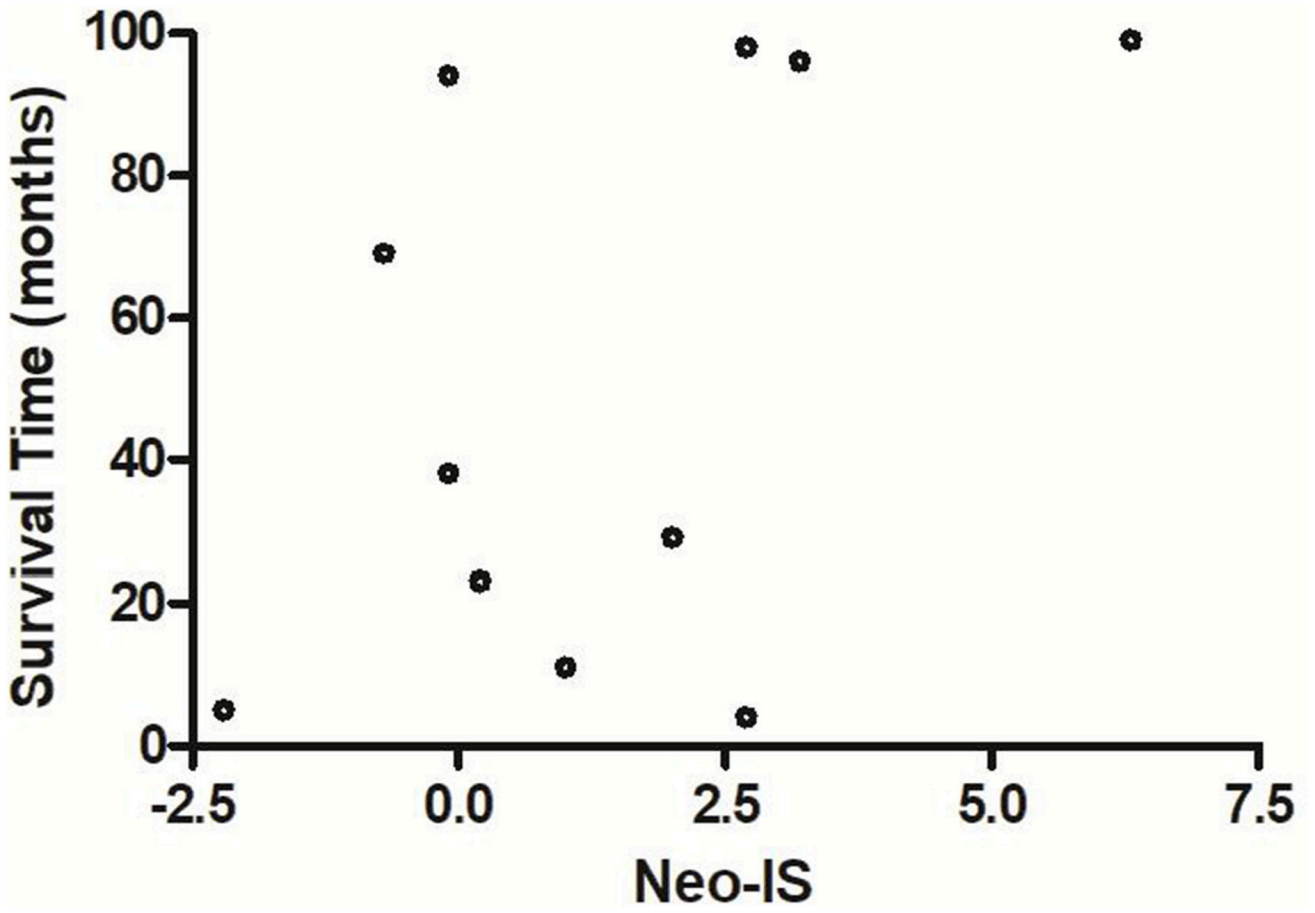
Survival time of different neo-IS with high-risk RCC patients.

The OS of neo-IS_high_ RCC patients with low- and intermediate- risk was longer than that of neo-IS_low_ patients (*P* = 0.026) (*P* = 0.019) (Figures 9A,B). With the limited cases of high-risk patients, we found that median survival time of six neo-IS_high_ RCC patients was longer than those of neo-IS_low_ patients (Figure 10).

## Discussion

Although developments in diagnostic imaging techniques have enabled the early detection of RCC, 20–30% of patients treated for localized RCC will experience tumor recurrence and metastasis after surgical resection (20). The high rates of tumor recurrence and metastasis emphasize the significance of postoperative surveillance. The nucleolar grading system and pTNM staging are prognostic factors validated by the International Society of Urological Pathology (ISUP) consensus (21). The various outcomes of RCC patients require an accurate prognostic model to guide the follow-up. These models are mostly composed of clinical factors and pathological features. The UISS, which divides localized RCC into three grades, is used frequently at present (4). Therefore, we analyzed the prognostic value of TNM staging, risk stratification, pathological features, and other clinical factors in RCC patients. We found that platelets, ECOG, staging, and risk stratification played roles in the prognosis of localized RCC patients in univariate analysis.

Although the TNM staging system, risk stratification, and other clinicopathological indicators were not considered with host immunity, increasing evidence suggests that an IS including CD3-positive and CD8-positive cell densities in TC and IM had a prognostic value to supplement the TNM staging system (7). In order to promote the use of the IS proposed by the SITC in clinics, an international task was launched (22). In our study, RCC patients with a higher IS in that proposed by the SITC had a better survival compared with patients who had a lower IS. This finding is consistent with that of the prognostic value of the IS proposed by the SITC in gastrointestinal and other cancers.

The IS proposed by the SITC provides a score ranging from 0 (I0), with low CD3 and CD8 cell densities found in the CT and IM, to 4 (I4), with high densities of both cell types in both regions. Although the IS proposed by the SITC includes CD3 and CD8 in the CT and IM, the roles of different cell types in different regions are uncertain. So, we built a neo-IS based on densities of immune cells in the CT and IM, respectively.

The CD3 antigen is a pan-T cell marker which is expressed on all T cells and comprises the T cell receptor as a protein complex. The densities of CD3^+^ tumor-infiltrating lymphocytes might reflect ongoing immunity against tumor cells and has been attributed to positive outcomes in breast cancers (23). Total T lymphocyte (CD3) densities in the CT were also significantly correlated with survival in colorectal cancer patients (24). We also found CD$3_{\text{CT}}^{+}$ T cells to be a protective factor in RCC patients (Table 3), which is consistent with the findings in other cancers. Immune checkpoints on infiltrating T cells are key regulators of immune escape in cancers. In recent years, studies on immune checkpoint molecules including PD-1/PD-L1 have attracted increasing attention. PD-1 is a member of B7 family that regulates T cell antigen-specific receptor signaling. PD-L1 binds to PD-1 to deliver an inhibitory signal to T cells and functions as a negative regulator of immunity. PD-L1 expressed on tumor cells or lymphocytes was associated with poor survival in RCC patients and PD-L1 expressed on activated T cells down-regulated primed T cells responses (25). However, we found that CD3^+^PD-L$1_{\text{IM}}^{+}$ played a positive role (β −0.012, *P* = 0.039 *P* < 0.05) in univariate analysis but it was removed from the multivariate regression analysis for its correlation with CD$3_{\text{CT}}^{+}$. Our finding suggests that CD3^+^ cells in the tumor microenvironment still affect important survival benefits despite partial disturbance of the anti-tumor immunity of CD3^+^ cells by PD-L1 co-expression.

A number of studies have shown that increased CD8+ T cell infiltration was related to a good prognosis in many cancers (26); however, several exceptions have emerged in RCC. Primary studies on RCC showed that PD-1 expression on immune cells was associated with a poor clinical outcome (27) and infiltration of intratumoral PD-1+ T cells was an independent adverse predictor of survival (28), whereas our study observed that CD8^+^PD-$1_{\text{IM}}^{+}$, which had a negative effect on survival in RCC, remained in the neo-IS due to its correlation with CD8^+^PD-$1_{\text{CT}}^{+}$ (Figure 4D).Its role may be due to the inhibition of tumor immunity by PD-1^+^CD$8_{\text{IM}}^{+}$ immune cells (29).

CD68^+^ macrophages are innate immune cells that play a broad role in host defense and the maintenance of tissue homeostasis, but some research had shown that increased CD68^+^ macrophage densities were related to increased tumor progression and worse prognosis in RCC patients (30). In univariate analysis, we also found that high CD$68_{\text{IM}}^{+}$ macrophage densities were a factor (β 0.003, *P* = 0.033 < 0.05) associated with the prognosis of RCC patients. This finding suggests that CD$68_{\text{IM}}^{+}$ macrophages may be favorable for the immunosuppressive M2 phenotype in RCC. However, CD$68_{\text{IM}}^{+}$ was removed from the neo-IS for its correlation with PD-1^+^CD$8_{\text{IM}}^{+}$. IDO is an enzyme that catalyzes the degradation of the amino acid tryptophan and plays a critical role in immunosuppressive mechanisms. The expression and localization of IDO in the tumor microenvironment are diverse, including tumor cells and immune cells (31). However, one study reported IDO-1 expression to be totally absent in tumor cells and only present in a few macrophages, while its expression was positively correlated with CD8+ T cell expression (32). We used CK to label tumor epithelial cells. Similarly, we found no expression of IDO^+^CK^+^ cells in our study. Nevertheless, we found that the low co-expression of IDO^+^CD8^+^ or IDO^+^CD68^+^ in RCC had no predictive value for RCC prognosis.

Treg, characterized by expression of the forkhead family transcription factor T Foxp3, are essential components of homeostasis in the immune system, which inhibits the functions of differentiated CD4+ and CD8+ T cells and activities of B cells, macrophages, and natural killer cells (33). High peritumoral levels of Tregs predicted deleterious outcomes in RCC, while Tregs in intratumoral areas had no prognostic value in RCC (34). However, in our study, CD4^+^Foxp$3_{\text{IM}}^{+}$ and CD4^+^Foxp$3_{\text{CT}}^{+}$ had no prognostic value in RCC. TNFR2 is a member of the TNFR family, also known as the TNFR superfamily. TNFR2 has two types, membrane-binding TNFR2 (mTNFR2) and soluble TNFR2 (sTNFR2) (35). Treg cells expressing TNFR2 is the maximally suppressive subgroup of Treg in humans. TNFR2^+^Treg contributed to cervical cancer development (36) but had no prognostic value in RCC in our study. In humans, CD45RO^+^ are thought to be memory T cells. Memory T cells are generated during antigen-mediated immune responses and survive for a long time even in the absence of antigens in the peripheral tissues. Primary studies found that increasing infiltration of CD45RO^+^ lymphocytes was correlated with increased survival in colorectal and gastric tumor immunity (37), but RCC patients with low CD45RO^+^ T cell densities had a significantly better prognosis than that in patients with high densities (38). In our study, we also found no prognostic value for CD45RO in RCC. However, memory Treg (mTreg) cells, which reside in tissues after the elimination of antigens, have a high immunosuppressive capacity and decreased proliferative index (14, 39). The results of our study suggested that a high density of mTreg (CD4^+^Foxp3^+^CD45RO^+^)_CT_ in RCC patients predicted a poor prognosis.

The neo-IS was constructed using three features; namely, CD3, CD4^+^Foxp3^+^CD45RO^+^, and CD8^+^PD-1^+^ and included immunosuppression-related factors such as memory Treg cells and immune-checkpoint receptor positive CD8 T cells. The neo-IS not only included more biomarkers but also analyzed specific cell subsets by multiplex fluorescent IHC in the CT and IM, including CD4^+^Foxp3^+^CD45RO^+^ in the CT and CD8^+^PD-1^+^ in the IM. We found that the AUC of the neo-IS was higher than that of the IS proposed by the SITC. Therefore, the neo-IS was more precise and comprehensive than the IS proposed by the SITC in our study.

Risk stratification and neo-IS were independent factors for the prognosis of RCC patients in our study. Moreover, a lower neo-IS suggested a worse outcome in RCC patients with low- and intermediate- risk stratification. Hence, the neo-IS may supplement the prognostic value of risk stratification to guide surveillance and subsequent therapy. It suggests that we should follow-up more frequently with neo-IS_low_ RCC patients of low- and intermediate- risk than neo-IS_high_ RCC patients *to* find the recurrence of patients earlier. Meanwhile, the neo-IS may be useful to guide further immunotherapy such as anti-PD-1 for recurrent patients. The present study of neo-IS was based on a relatively small cohort of 82 patients from a single center. A large-scale and multi-center perspective study is planned to further validate the neo-IS.

## Data Availability

All datasets generated for this study are included in the manuscript and/or the supplementary files.

## Ethics Statement

This research project was approved by the Ethics Committee of Tianjin Cancer Institute and Hospital. Written consents were obtained from each patient.

## Consent for Publication

Written consents were obtained from each patient to publishing their pathological images as represent Figures.

## Author Contributions

CG, FW, and XR analyzed and interpreted the patient data. CG, HZ, and SB performed the multiplex fluorescent immunohistochemistry (IHC) staining and multispectral imaging of the cancer tissues. CG, YW, and ZY participated statistical analysis. CG was a major contributor in writing the manuscript. XR and FW interpreted and revised the manuscript. All authors read and approved the final manuscript.

### Conflict of Interest Statement

The authors declare that the research was conducted in the absence of any commercial or financial relationships that could be construed as a potential conflict of interest.
